# Hypofractionated radiotherapy in locally advanced bladder cancer: an individual patient data meta-analysis of the BC2001 and BCON trials

**DOI:** 10.1016/S1470-2045(20)30607-0

**Published:** 2021-02

**Authors:** Ananya Choudhury, Nuria Porta, Emma Hall, Yee Pei Song, Ruth Owen, Ranald MacKay, Catharine M L West, Rebecca Lewis, Syed A Hussain, Nicholas D James, Robert Huddart, Peter Hoskin, BC2001 and BCON investigators

**Affiliations:** a Division of Cancer Sciences, University of Manchester, Manchester, UK; b Clinical Trials and Statistics Unit, The Institute of Cancer Research, London, UK; c Radiotherapy and Imaging Division, The Institute of Cancer Research, London, UK; d Department of Clinical Oncology, The Christie NHS Foundation Trust, Manchester, UK; e Department of Medical Physics and Engineering, The Christie NHS Foundation Trust, Manchester, UK; f Department of Medical Statistics, London School of Hygiene & Tropical Medicine, London, UK; g Department of Oncology and Metabolism, University of Sheffield, Sheffield, UK; h University Hospitals Birmingham NHS Foundation Trust, Birmingham, UK; i Royal Marsden NHS Foundation Trust, London, UK; j Mount Vernon Cancer Centre, Northwood, UK

## Abstract

**Background:**

Two radiotherapy fractionation schedules are used to treat locally advanced bladder cancer: 64 Gy in 32 fractions over 6·5 weeks and a hypofractionated schedule of 55 Gy in 20 fractions over 4 weeks. Long-term outcomes of these schedules in several cohort studies and case series suggest that response, survival, and toxicity are similar, but no direct comparison has been published. The present study aimed to assess the non-inferiority of 55 Gy in 20 fractions to 64 Gy in 32 fractions in terms of invasive locoregional control and late toxicity in patients with locally advanced bladder cancer.

**Methods:**

We did a meta-analysis of individual patient data from patients (age ≥18 years) with locally advanced bladder cancer (T1G3 [high-grade non-muscle invasive] or T2–T4, N0M0) enrolled in two multicentre, randomised, controlled, phase 3 trials done in the UK: BC2001 (NCT00024349; assessing addition of chemotherapy to radiotherapy) and BCON (NCT00033436; assessing hypoxia-modifying therapy combined with radiotherapy). In each trial, the fractionation schedule was chosen according to local standard practice. Co-primary endpoints were invasive locoregional control (non-inferiority margin hazard ratio [HR]=1·25); and late bladder or rectum toxicity, assessed with the Late Effects Normal Tissue Task Force-Subjective, Objective, Management, Analytic tool (non-inferiority margin for absolute risk difference [RD]=10%). If non-inferiority was met for invasive locoregional control, superiority could be considered if the 95% CI for the treatment effect excluded the null effect (HR=1). One-stage individual patient data meta-analysis models for the time-to-event and binary outcomes were used, accounting for trial differences, within-centre correlation, randomised treatment received, baseline variable imbalances, and potential confounding from relevant prognostic factors.

**Findings:**

782 patients with known fractionation schedules (456 from the BC2001 trial and 326 from the BCON trial; 376 (48%) received 64 Gy in 32 fractions and 406 (52%) received 55 Gy in 20 fractions) were included in our meta-analysis. Median follow-up was 120 months (IQR 99–159). Patients who received 55 Gy in 20 fractions had a lower risk of invasive locoregional recurrence than those who received 64 Gy in 32 fractions (adjusted HR 0·71 [95% CI 0·52–0·96]). Both schedules had similar toxicity profiles (adjusted RD −3·37% [95% CI −11·85 to 5·10]).

**Interpretation:**

A hypofractionated schedule of 55 Gy in 20 fractions is non-inferior to 64 Gy in 32 fractions with regard to both invasive locoregional control and toxicity, and is superior with regard to invasive locoregional control. 55 Gy in 20 fractions should be adopted as a standard of care for bladder preservation in patients with locally advanced bladder cancer.

**Funding:**

Cancer Research UK.

## Introduction

Bladder preservation therapy is an alternative to surgery for the management of locally advanced bladder cancer. Typically, this strategy comprises pretreatment staging with transurethral resection of the tumour and cross-sectional imaging, followed by radiotherapy with or without a radiosensitiser. Radiotherapy might also be preceded by neoadjuvant chemotherapy. Combining radiotherapy with a radiosensitiser gives similar rates of disease-specific survival and overall survival (about 50% at 5 years for both endpoints) compared with surgery.^1^, ^2^, ^3^, ^4^ The two largest phase 3, randomised, controlled trials of bladder preservation showed benefit in terms of locoregional disease-free survival (the BC2001 trial^3^, ^5^) and overall survival (BCON^1^) with use of chemotherapy (BC2001) or hypoxia-modifying therapy (BCON) concurrent with radiotherapy, compared with radiotherapy alone.

Both trials permitted two commonly used radiotherapy fractionation schedules, 64 Gy in 32 fractions over 6·5 weeks and a hypofractionated schedule of 55 Gy in 20 fractions over 4 weeks. Although no direct comparisons of these schedules have been made previously, published case series and cohort studies suggest that outcomes and late toxicity are similar.^6^ The present study aimed to assess whether 55 Gy in 20 fractions is non-inferior to 64 Gy in 32 fractions in terms of invasive locoregional control and late bladder and bowel toxicity, by assessing combined individual patient data from the BC2001 and BCON randomised, phase 3 trials.

**Research in context**

**Evidence before this study**

Before this individual patient data meta-analysis was initiated (Jan 1, 2019), to our knowledge no published randomised controlled trials or meta-analyses comparing the two most common radiotherapy dose and fraction schedules used in muscle-invasive bladder cancer were available. Both 64 Gy in 32 fractions and a hypofractionated schedule of 55 Gy in 20 fractions are used as standard treatment in the UK and were part of the protocols in the BC2001 and BCON phase 3, randomised, controlled trials. We searched PubMed using the terms (hypofractionated radiotherapy AND muscle-invasive bladder cancer) AND (loco-regional control) AND (overall survival) for clinical trials and meta-analyses published up to May 31, 2020, without language restrictions. We identified no studies directly comparing the two schedules, although published series suggested that outcome and late toxicity were similar.

**Added value of this study**

To our knowledge, this study is the first published individual patient data meta-analysis comparing outcomes from the two most commonly used radiotherapy schedules for muscle-invasive bladder cancer. We aimed to confirm that moderately hypofractionated radiotherapy with 55 Gy in 20 fractions over 4 weeks was non-inferior to 64 Gy in 32 fractions over 6·5 weeks for invasive locoregional control at 5 years. This study provides evidence that moderately hypofractionated radiotherapy was indeed non-inferior with respect to invasive locoregional control and late bladder and rectum toxicity, and significantly improved invasive locoregional control. The observed benefit was robust regardless of radiosensitisation or radiosensitiser.

**Implications of all the available evidence**

With these findings, 55 Gy in 20 fractions over 4 weeks should be considered as the new standard of care for bladder preservation therapy in patients with muscle-invasive bladder cancer.

## Methods

### Study design and participants

We did a meta-analysis of individual patient data from patients with invasive bladder cancer enrolled in the multicentre, randomised, controlled, phase 3 trials BC2001^3^, ^5^ and BCON.^1^ The trials, including full eligibility criteria, procedures, and statistical analysis plans, have been previously reported. Inclusion and exclusion criteria were similar between the trials (appendix p 1). Briefly, BC2001 (NCT00024349) was a phase 3, randomised trial.^3^, ^5^ 458 patients (age ≥18 years) from45 UK National Health Service (NHS) radiotherapy departments with a diagnosis of transitional cell bladder carcinoma (stage T2–T4N0M0) suitable for treatment with radical radiotherapy were recruited. Patients were randomly assigned with a partial two-by-two factorial design (1:1 in each randomisation) to receive either radiotherapy alone or radiotherapy with concomitant chemotherapy (fluorouracil [500 mg/m^2^ body surface area per day on days 1–5 and days 16–20] and mitomycin C [12 mg/m^2^ on day 1]); and either standard whole-bladder radiotherapy or reduced high-dose volume radiotherapy with tumour boost.

BCON (NCT00033436) was a phase 3, randomised trial with parallel design.^1^ 333 patients (age ≥18 years) from 13 UK NHS radiotherapy departments (of which eight were common to both trials) with a diagnosis of transitional cell bladder carcinoma (stages T1G3N0M0 [high-grade non-muscle invasive] to T4aN0M0) were recruited. Patients were randomly assigned (1:1) to radiotherapy with or without hypoxia modification with carbogen (2% CO_2_ and 98% O_2_ at 15 L/min for 5 min before and during each fraction) and nicotinamide (orally at 40–60 mg/kg, given 1·5–2·0 h before each fraction). All patients in the BCON trial received standard whole bladder radiotherapy.

In both trials, fractionation schedule for radiotherapy (64 Gy in 32 fractions over 6·5 weeks or 55 Gy in 20 fractions over 4 weeks) was chosen by each participating centre according to local standard practice. Radiotherapy was delivered with a conventional or 3D conformal technique when the bladder was empty. An expansion of 1·5 cm was used from clinical target volume to planned target volume. Pelvic lymph nodes were not included in the clinical target volume. Generally, all patients at the same site were treated with the same fractionation schedule, but with exceptions at six sites (four sites in BC2001 and two sites in BCON). Initial staging was ascertained in both trials by cystoscopic examination and biopsy to confirm histological diagnosis, CT or MRI of the abdomen and pelvis, and chest radiography (chest CT also allowed in BC2001).

Tumour control was assessed in BC2001 by means of physical examination, chest radiography, and rigid or flexible cystoscopy at 6, 9, and 12 months after randomisation, and then annually. Biopsy of the tumour bed and normal bladder was mandated at 6 months and repeated as indicated at subsequent cystoscopies. CT of the abdomen and pelvis was done at 1 year and 2 years after randomisation and then as indicated. In BCON, cystoscopic examination occurred 6 months after radiotherapy, and every 6 months for up to 5 years; CT and upper tract endoscopy were done when indicated from cystoscopy. Management of patients who relapsed was according to local site practice in both trials.

In BC2001, annual follow-up for disease events (recurrence of local or distant disease) and patient status was prospectively collected up to July 11, 2016. In BCON, recruiting sites were contacted in 2018 to obtain long-term survival data (recurrence of local or distant disease and patient status), with a data lock on Oct 31, 2018.

Both trials measured late toxicity up to 5 years after the end of radiotherapy with the Late Effects Normal Tissue Task Force (LENT)-Subjective, Objective, Management, Analytic (SOMA)^7^, ^8^ tool. In BCON, only urinary and rectal dysfunction subscales were recorded, and they were assessed more frequently (every 3 months in year 1 and every 6 months in years 2–5) than in BC2001 (every 3 months in year 1, and annually thereafter). In the BC2001 trial, health-related quality of life (HRQOL) was assessed at the end of treatment, 6 months and 12 months after randomisation, and then annually up to 5 years with the Functional Assessment of Cancer Therapy-Bladder (FACT-BL) module.^9^ A similar HRQOL schedule was planned in BCON, but data return was sparse and analysis was not pursued.

Details on the key features and endpoints of both trials are summarised in the appendix (p 2).

### Statistical analysis

Based on information available in both trials, we defined common endpoints for the meta-analysis. The co-primary endpoints were invasive locoregional control, defined as the rate of control of invasive bladder recurrence or recurrence in pelvic nodes (ie, invasive locoregional recurrence), and late rectum or bladder toxicity.

The timepoint of interest for the invasive locoregional control estimate was 3 years. To account for the difference in length of disease follow-up assessments between trials, the period of observation was set at 5 years. Patients were therefore censored at 5 years if known to be alive and disease-free; at last known disease assessment if alive and disease-free with less than 5 years follow-up; at the date of distant recurrence (unless invasive locoregional recurrence was diagnosed within 30 days after diagnosis of distant recurrence, to account for delay in confirming diagnoses); at the date of diagnosis of a second primary tumour (only collected in BC2001); or at the date of death due to any cause (if recurrence free).

Late toxicity was measured by the proportion of patients who had a grade 3–4 rectum or bladder adverse event as assessed by the LENT-SOMA scale, during the 5 years after randomisation.

The secondary endpoint was overall survival, defined as time from the date of randomisation to the date of death due to any cause. Patients alive at their last known follow-up were censored. All follow-up periods available in either trial were used for this endpoint. As a post-hoc endpoint of our meta-analysis, we evaluated bladder cancer-specific survival, defined as time from randomisation to death due to bladder cancer; patients who died due to other causes were censored at their date of death. A further post-hoc exploratory endpoint was change from baseline in HRQOL (BC2001 only).

Individual patient data were combined into one dataset. All patients in the BCON and BC2001 trials who received at least one fraction of radiotherapy and for whom the fractionation schedule was known were included in the meta-analysis. Given that the fractionation schedules were not randomised and confounding was likely to occur, a one-stage individual patient data meta-analysis approach was chosen due to its flexibility to adjust for potential confounders^10^, ^11^, ^12^ while preserving clustering within each trial.^13^ As trials differed in baseline data collection, this affected the confounders we could adjust for. We explored sex, age, allocation to radiosensitiser and reduced high-dose volume radiotherapy interventions, tumour stage and grade, extent of resection, use of neoadjuvant chemotherapy, and haemoglobin, which were collected in both trials. Imbalance in baseline characteristics was investigated with standardised differences,^14^, ^15^ which provided a common scale (%) for the magnitude of imbalance between fractionation groups for all baseline variables. Any variables with a greater than 10% standardised difference in the combined dataset were considered potential confounders and investigated in the meta-analysis.

We hypothesised that 55 Gy in 20 fractions would be non-inferior to 64 Gy in 32 fractions in terms of disease control and late toxicity. For each endpoint, non-inferiority would be declared if the upper limit of the 95% CI of the estimated fractionation differences was smaller than the non-inferiority margin. The prespecified non-inferiority margin for invasive locoregional control was a hazard ratio (HR) of HR_non-inferiority_=1·25, and for late bladder and rectum toxicity, an absolute risk difference (RD) of RD_non-inferiority_=10%. Statistical significance was assessed with 95% CIs, with 5% significance corresponding to the null hypothesis value being outside the 95% CI. If non-inferiority was met for invasive locoregional control, superiority could be considered if the 95% CI for the treatment effect excluded the null effect (HR=1).

For the time-to-event endpoints, a crude analysis to estimate the hazard ratio (HR) representing the relative difference between fractionation schedules was first done by fitting a stratified Cox proportional hazards model with fractionation schedule as the predictor, a frailty term for site clustering, and stratifying by trial. An adjusted HR (aHR) for fractionation effect was fitted similarly, but incorporating trial intervention (allocated use of concurrent radiosensitiser), prespecified prognostic factors, and any potential confounder with baseline imbalance (leading to >10% variation in the crude fractionation effect when the potential confounder was added to the model) or showing univariable association (at the 5% level) with the endpoint. As in the BC2001 trial, prespecified prognostic factors for invasive locoregional control were age, sex, tumour stage, use of neoadjuvant chemotherapy, and extent of resection; and for overall survival were age and sex.^16^ Model assumptions were assessed by graphical assessment of residuals. A likelihood ratio test for heterogeneity of fractionation effect across trials was done by considering an extended model including the interaction of fractionation schedule and trial.

For the analysis of grade 3–4 rectum and bladder toxicities within 5 years, toxicities reported within 3 months before first recurrence or death related to bladder cancer were treated as missing to avoid interpreting recurrence symptoms as toxicities. As a sensitivity analysis, we present the results when such censoring was not done. The absolute RD between fractionation schedules in having grade 3–4 rectum or bladder toxicity over 5 years was estimated with a generalised linear binomial model with a random intercept for treatment site, to account for clustering within sites.^17^ A crude model was first fitted for fractionation schedule including trial as a fixed effect. In the adjusted analysis, we also included trial intervention, age, sex, and any potential confounder with baseline imbalance (leading to >10% variation in the crude fractionation relative effect) or showing univariable association (at the 5% level) with the toxicity outcome. Each model was adjusted on the subset of patients with available values for variables in the model. Heterogeneity between trials was assessed by considering an interaction effect between fractionation schedule and trial.

In subgroup analyses, we explored the fractionation effect within trials and in patients who received radiotherapy alone for invasive locoregional control, overall survival, and toxicity to assess robustness of results; aHR was the output with adjustment for the same factors as in the primary analyses. To investigate the effect of fractionation schedule on HRQOL in the BC2001 trial only, we employed similar methods as used for the trial's HRQOL substudy, with mean change from baseline in FACT-BL (including TOTAL and subscale) scores summarised at each timepoint.^18^ Only patients with paired baseline and follow-up data were included in the analysis. A 1% significance level and corresponding 99% CIs were considered to account for multiple timepoints and subscales of interest.^18^ Our post-hoc analysis of bladder-cancer specific survival is outlined in the appendix (p 9).

Forest plots of fractionation effects on each outcome were used to graphically assess the degree of overlap between the 95% CIs of each trial. Data were analysed with Stata (version 15.0) and the R (version 3.6.0) survival^19^ and geepack^20^ packages. Analysis was based on a data snapshot on July 11, 2016, for BC2001 and Oct 1, 2018, for BCON.

Expanded details of our statistical methods are provided in the appendix (pp 3–4).

### Role of the funding source

The funder of the study had no role in study design, data collection, data analysis, data interpretation, or writing of the report. All authors had full access to all data in the study and had final responsibility for the decision to submit for publication.

## Results

**Figure 1:**
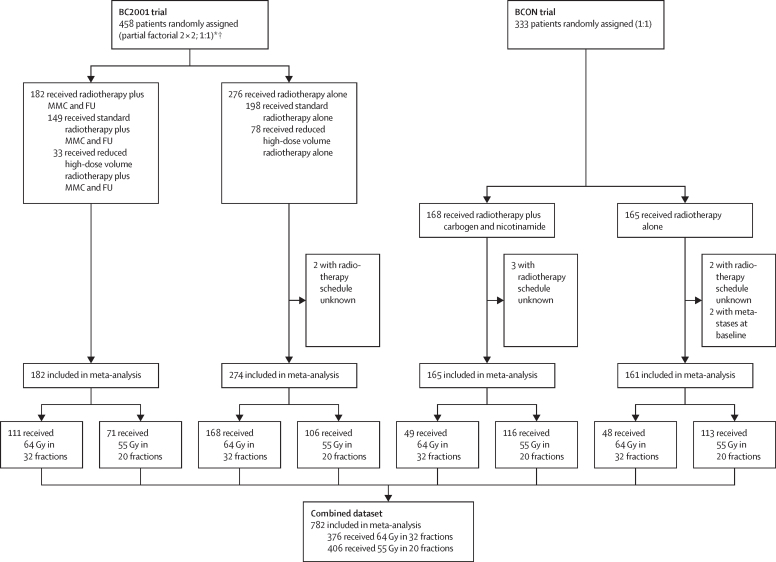
Trial profiles and final dataset MMC=mytomicin C. FU=fluorouracil. *98 excluded from chemotherapy randomisation (radiotherapy plus chemotherapy *vs* radiotherapy alone): 53 ineligible for chemotherapy; 34 withdrew or were withdrawn by physician; four had other reasons; and seven had unknown reasons. †239 excluded from radiotherapy randomisation (standard radiotherapy *vs* reduced high-dose volume radiotherapy): 84 entered the trial after radiotherapy randomisation closed; 54 at centres not participating in radiotherapy randomisation; 47 with multiple tumours; 44 withdrew or were withdrawn by physician; and ten with administrative or unknown reasons.

Of 458 patients in the BC2001 trial, 456 were included in the meta-analysis (fractionation schedule was unknown for two patients); 279 (61%) received 32 fractions and 177 (39%) received 20 fractions. Median follow-up was 118 months (IQR 100–137). Of 333 patients in the BCON trial, 326 were included in the meta-analysis (fractionation schedule was unknown for five patients and two further patients were found to have metastases at baseline); 97 (30%) received 32 fractions and 229 (70%) received 20 fractions. Median follow-up was 159 months (IQR 91–181). Our combined dataset therefore consisted of 782 patients of whom 376 (48%) received 32 fractions and 406 (52%) received 20 fractions (figure 1). Median follow-up in the combined dataset was 120 months (IQR 99–159).

Patient characteristics in the combined dataset showed imbalances between fractionation groups (table 1) with respect to tumour stage, tumour grade, and extent of resection. The imbalance in the proportion receiving reduced high-dose volume radiotherapy in BC2001 reflects the partial two-by-two design and the fact that the radiotherapy randomisation closed first.^5^

**Table 1:** Baseline characteristics

		**BC2001 trial**			**BCON trial**			**Combined dataset**		
		64 Gy in 32 fractions (n=279)	55 Gy in 20 fractions (n=177)	Standardised difference, %	64 Gy in 32 fractions (n=97)	55 Gy in 20 fractions (n=229)	Standardised difference, %	64 Gy in 32 fractions (n=376)	55 Gy in 20 fractions (n=406)	Standardised difference, %
Sex										
	Female	50 (18%)	37 (21%)	..	17 (18%)	48 (21%)	..	67 (18%)	85 (21%)	..
	Male	229 (82%)	140 (79%)	7·5%	80 (82%)	181 (79%)	8·7%	309 (82%)	321 (79%)	7·9%
Age, years		71·4 (8·7)	71·6 (7·9)	1·3%	72·6 (7·6)	73·0 (7·8)	−5·4%	71·7 (8·4)	72·4 (7·8)	7·9%
Radiotherapy plus radiosensitiser		111 (40%)	71 (40%)	0·7%	49 (51%)	116 (51%)	0·3%	160 (43%)	187 (46%)	7·1%
Reduced high-dose volume radiotherapy		75 (27%)	35 (20%)	16·9%	0	0	..	75 (20%)	35 (9%)	32·8%
Tumour stage										
	1	1* (<1%)	0	..	13 (13%)	17 (7%)	..	14 (4%)	17 (4%)	..
	2	251 (90%)	129 (73%)	..	68 (70%)	147 (64%)	..	319 (85%)	276 (68%)	..
	3	20 (7%)	40 (23%)	..	14 (14%)	54 (24%)	..	34 (9%)	94 (23%)	..
	4	7 (3%)	8 (5%)	47·6%	2 (2%)	10 (4%)	32·1%	9 (2%)	18 (4%)	42·6%
	Unknown	0	0	..	0	1	..	0	1	..
Tumour grade										
	1	1 (<1%)	0	..	0	0	..	1 (<1%)	0	..
	2	40 (14%)	19 (11%)	..	16 (16%)	30 (13%)	..	56 (15%)	49 (12%)	..
	3	236 (85%)	156 (89%)	13·9%	81 (84%)	198 (87%)	9·4%	317 (85%)	354 (88%)	11·1%
	Unknown	2	2	..	0	1	..	2	3	..
Extent of resection										
	Biopsy or not resected	24 (9%)	23 (13%)	..	25 (26%)	62 (28%)	..	49 (13%)	85 (22%)	..
	Complete	175 (63%)	81 (47%)	..	46 (47%)	83 (38%)	..	221 (59%)	164 (42%)	..
	Partial	77 (28%)	70 (40%)	34·4%	26 (27%)	73 (33%)	19·6%	103 (28%)	143 (36%)	36·1%
	Unknown	3	3	..	0	11	..	3	14	..
Neoadjuvant chemotherapy		65 (23%)	69 (39%)	34·4%	0	0	..	65 (17%)	69 (17%)	0·8%
Haemoglobin, g/dL†		13·1 (1·8)	12·6 (1·8)	27·4%	13·8 (1·7)	13·6 (1·6)	11·3%	13·2 (1·8)	13·1 (1·8)	5·7%

Data are n (%), mean (SD), or n. Standardised difference represents the difference in means or proportions divided by its standard error; it is therefore a measure of the average difference between groups expressed in standard deviation units. A difference greater than 10% expresses that the observed difference between fractionation groups is more than 10% of the observed variability. Percentages were calculated for total number of patients without missing values.

* This tumour was deemed to be pathological stage T1, but radiological staging confirmed the tumour as T3 and the patient was considered to be eligible for the trial.

† Unknown in four patients receiving 64 Gy in 32 fractions (one in BC2001 and three in BCON) and two patients receiving 55 Gy in 20 fractions (BCON).

**Figure 2:**
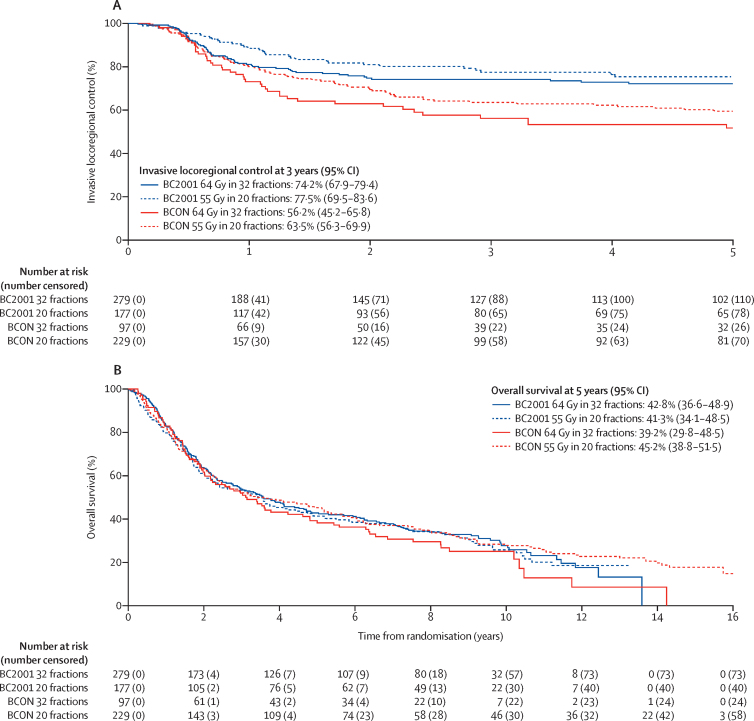
Kaplan-Meier estimates of observed invasive locoregional control (A) and observed overall survival (B) by trial and fractionation group

**Figure 3:**
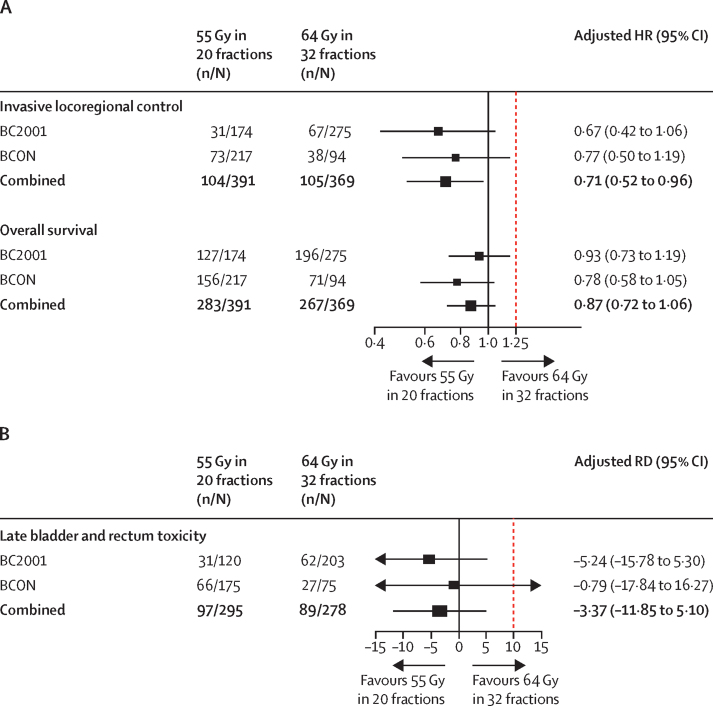
Forest plots of the fractionation effect of 64 Gy in 32 fractions versus 55 Gy in 20 fractions for invasive locoregional control and overall survival (A) and toxicity (B) Invasive locoregional control combined HR estimates adjusted for age, sex, randomised treatment, extent of resection, tumour stage, haemoglobin, and neoadjuvant chemotherapy. Overall survival combined HR estimates adjusted for age, sex, randomised treatment, extent of resection, tumour stage, and haemoglobin. Invasive locoregional control and overall survival were modelled on random effects for treatment site and stratified by trial. Late rectum or bladder toxicity combined absolute RD estimates adjusted for age, sex, randomised treatment, and trial, with a random-effects model for treatment site. Models were adjusted on the subset of patients with available values for variables in the model. Red dotted line represents the non-inferiority margin. HR=hazard ratio. RD=risk difference.

218 (28%) of 782 patients had invasive locoregional recurrence within 5 years: 106 (28%) of 376 receiving 32 fractions and 112 (28%) of 406 receiving 20 fractions. Median follow-up in our analysis of invasive locoregional control was 60 months (IQR 21–60). Invasive locoregional control rates over time per trial and fractionation group are summarised in figure 2A. In the combined dataset, crude one-stage meta-analysis gave an HR of 0·83 (95% CI 0·63–1·10) for patients receiving 20 fractions versus those receiving 32 fractions (appendix p 5). After accounting for age, sex, trial intervention, extent of resection, tumour stage, haemoglobin, and use of neoadjuvant chemotherapy, the 20 fraction schedule had a lower hazard than the 32 fraction schedule (aHR 0·71 [95% CI 0·52–0·96]; figure 3A; appendix pp 5–6). As the upper limit of the 95% CI for both the crude and adjusted estimates was lower than the prespecified HR_non-inferiority_ (1·25), non-inferiority of the 20 fractions schedule could be concluded. The estimated fractionation effect within subgroups of treatment intervention is shown in the appendix (p 5). No significant heterogeneity across trials was found (χ^2^=0·066, p=0·80).

571 (73%) of 782 patients died during follow-up, 273 (73%) of 376 who received 32 fractions and 298 (73%) of 406 who received 20 fractions. Overall survival rates over time per trial and fractionation group are shown in figure 2B. Crude one-stage meta-analysis of overall survival gave an HR of 0·99 (95% CI 0·83–1·18) for patients who received 20 fractions versus those who received 32 fractions (appendix p 7). After accounting for age, sex, trial intervention, extent of resection, tumour stage, and haemoglobin, the aHR was 0·87 (95% CI 0·72–1·06; figure 3A; appendix pp 7–8). The estimated fractionation effect within subgroups of treatment intervention for overall survival is shown in the appendix (p 7). The likelihood ratio test indicated some heterogeneity in fractionation effect across trials (χ^2^=5·37, p=0·02), as shown by fractionation effect being larger in the BCON trial (figure 3A). The post-hoc analysis of bladder cancer-specific survival is shown in the appendix (p 9).

Bladder and rectum LENT-SOMA toxicity data in the BC2001 trial were available for analysis in 203 (73%) of 279 patients receiving 32 fractions and 120 (68%) of 177 receiving 20 fractions. In the BCON trial, data were available in 75 (77%) of 97 patients receiving 32 fractions and 175 (76%) of 229 receiving 20 fractions (table 2). In the combined dataset, 278 (74%) of 376 patients receiving 32 fractions and 295 (73%) of 406 receiving 20 fractions had toxicity data available. The appendix (p 10) shows the distribution of baseline variables over fractionation groups in patients with data available for toxicity analysis. The proportion of patients with grade 3–4 rectum or bladder toxicity within 5 years of radiotherapy treatment was similar between fractionation groups (table 2). The combined one-stage crude meta-analysis showed an RD of −2·88% (95% CI −11·15 to 5·39; appendix p 11) for 20 fractions versus 32 fractions. Similar results were obtained after adjusting age, sex, and trial intervention (adjusted RD [aRD] −3·37% [95% CI −11·85 to 5·10]; figure 3B; appendix pp 11–12). A similar difference in risk was found in a sensitivity analysis in which toxicities that occurred within 3 months of a recurrence event were not censored (aRD −3·82% [95% CI −11·88 to 4·24]). As the upper limit of both the crude and adjusted 95% CI in our main analyses was smaller than RD_non-inferiority_=10%, non-inferiority of 20 fractions compared with 32 fractions in relation to 5-year bladder and rectum toxicity could be concluded. The estimated fractionation effect within subgroups of treatment intervention for late toxicity is shown in the appendix (p 11). The test for interaction between trial intervention and fractionation group was significant (p=0·0086).

**Table 2:** LENT-SOMA grade 3–4 bladder or rectum toxicity after the end of treatment by fractionation groups

	**64 Gy in 32 fractions (n=278)**	**55 Gy in 20 fractions (n=295)**
**2-year late toxicity**		
Rectum	7 (3%)	17 (6%)
Bladder	66 (24%)	74 (25%)
Rectum or bladder	69 (25%)*	82 (28%)†
**5-year late toxicity**		
Rectum	8 (3%)	21 (7%)
Bladder	86 (31%)	88 (30%)
Rectum or bladder	89 (32%)‡	97 (33%)§

LENT-SOMA urinary and rectal dysfunction subscales were recorded up to 5 years after radiotherapy in the BC2001 and BCON trials. LENT-SOMA=Late Effects Normal Tissue Task Force-Subjective, Objective, Management, Analytic.

* Four patients with both.

† Nine patients with both.

‡ Five patients with both.

§ 12 patients with both.

In BC2001, baseline FACT-BL scores, as a measure of HRQOL, were balanced between fractionation schedules (appendix p 13). Although we observed a detrimental effect of 20 fractions versus 32 fractions at the end of treatment on the total score (estimated adjusted mean difference from baseline between fractionation groups −9·34 [99% CI −18·36 to −0·32], p=0·0077), this difference was not significant at 1 year (–1·29 [–12·31 to 9·72], p=0·76), nor at later times (appendix p 14).

The adjusted models of the combined dataset for invasive locoregional control, overall survival, and late toxicity (appendix pp 5, 7, 11) also provided estimates for the radiosensitiser effect. Significantly improved HRs were seen with a radiosensitiser compared with radiotherapy alone for invasive locoregional control (aHR 0·65 [95% CI 0·49–0·87]) and overall survival (aHR 0·83 [0·70–0·98]); and we observed no significant increase in late grade 3–4 rectum or bladder toxicity (aRD −1·40 [95% CI −9·43 to 6·63]).

## Discussion

To our knowledge, this study is the first to compare the outcomes of conventional fractionation with moderately hypofractionated radiotherapy for locally advanced bladder cancer. We found that hypofractionated radiotherapy with 55 Gy in 20 fractions was non-inferior to 64 Gy in 32 fractions in relation to invasive locoregional control and late bladder and rectum toxicity. Furthermore, since the 95% CI for the aHR estimate excluded a value of 1 (no effect), the adjusted analysis suggested that the 20 fraction schedule improved invasive locoregional control at the 5% level, despite patients treated with 55 Gy in 20 fractions having poor prognostic factors. Non-inferiority was also confirmed across intervention subgroups, regardless of whether a patient was treated with radiotherapy alone or radiotherapy with radiosensitisation. Moreover, if the same HR_non-inferiority_ (1·25) was considered for overall survival, the adjusted model would also suggest non-inferiority of the 20 fraction schedule. As such, there is a cogent argument for 55 Gy in 20 fractions being adopted as the standard of care in this patient group. Many studies from outside the UK advocate trimodality treatment with a complete transurethral resection of bladder being essential to undertake bladder preservation.^21^, ^22^, ^23^ Both the BC2001 and BCON trials had a high rate of local control despite high proportions of patients with incomplete resections. Although undertaking a complete transurethral resection of bladder might be optimal, the results of both trials suggest that bladder preservation can be achieved even in its absence.

Bladder cancer is considered a rapidly proliferating cancer, with a high α/β ratio of 10 Gy (and thus low fractionation sensitivity),^24^ and evidence suggests a loss of effective radiotherapy dose (γ radiation) of 0·2–0·4 Gy per day after approximately 5 weeks of treatment due to tumour cell repopulation (appendix p 15).^25^ In the linear-quadratic model to predict radiobiological response, use of an α/β ratio of 10 Gy without accounting for overall treatment time suggests that 64 Gy in 32 fractions and 55 Gy in 20 fractions have biologically effective doses (BEDs) of 76·8 Gy and 70·1 Gy. This difference is reduced when a time factor is included, with a maximum reduction for kick-off time (Tk; defined as the delay in tumour cell repopulation in response to radiotherapy) of 28 days or less. If BED was calculated with γ=0·36 and Tk=28 days, 64 Gy in 32 fractions and 55 Gy in 20 fractions have BEDs of 71·0 Gy and 70·1 Gy respectively. For both schedules to be equivalent without a time factor, an α/β ratio of 2 for bladder cancer would be required. Based on our fractionation effect estimates, tumour repopulation appears substantial with the longer 32-fraction regimen. Investigators have previously reported repopulation after 5 weeks.^25^, ^26^ Results on the effect of the 32-fraction regimen in this study suggest that repopulation occurs from as early as 4 weeks. Further data on outcomes from different radiotherapy schedules would be required to optimise a predictive model based on dose, fractionation schedule, and survival outcome. However, the finding that a moderately hypofractionated radiotherapy schedule is non-inferior for locally advanced bladder cancer is mostly probably due to a combination of an α/β ratio lower than 10 and a substantial effect of repopulation. The overall treatment time for rapidly proliferating cancers at high risk of repopulation is crucial, with evidence of detrimental outcomes when treatment is interrupted and prolonged. Guidelines are available for accommodating unexpected gaps in treatment.^27^

Enhanced acute toxicity is a concern with a shortened radiotherapy schedule,^28^ but, in the present study, differences in data collection between the trials limited our assessment of acute toxicity. HRQOL data from the BC2001 trial did suggest worse quality of life at the end of treatment with the hypofractionated schedule, but this did not result in excess treatment interruptions;^18^ and after 6 months no difference in HRQOL between radiotherapy schedules was seen.

Although concern is often expressed about the risk of late toxicity with hypofractionated radiotherapy, our meta-analysis showed no significant difference in late toxicity between fractionation regimens. Despite this finding, care should be taken when extrapolating these data to radiosensitisation with other treatments, such as immunotherapy, for which hypofractionation might have greater effect.^29^ Furthermore, no difference was observed in patient-reported HRQOL in the long-term after recovery from acute toxicity in the BC2001 trial. Published 5-year patient-reported outcomes showed excellent preservation of daily function with both fractionation schedules throughout the follow-up period.^18^ Our subgroup analysis of toxicity indicating a detrimental effect of 55 Gy in 20 fractions in patients receiving a concurrent radiosensitiser should be interpreted with caution, as any differences might relate to a combined benefit from the sensitiser and hypofractionation prolonging recurrence-free time, resulting in these patients having increased follow-up to collect toxicity data.

Innate challenges arise when combining data from two phase 3 trials with no preplanned meta-analysis. Acknowledging the limitations in this study, the primary outcome here differs from the primary endpoints in BC2001 (locoregional control, including non-muscle invasive bladder recurrences) and BCON (local relapse-free survival, including invasive recurrences and death). Given that the BCON dataset contains information on recurrence of muscle-invasive lesions only, the BC2001 trial secondary endpoint of invasive locoregional control was chosen as the primary endpoint for this meta-analysis as it could be defined in both trials. Furthermore, data collection differed between the trials, with toxicity data collected more frequently in the BCON trial than in the BC2001 trial. We overcame this issue by using cumulative reporting of adverse events over a common reporting period. BC2001 included prospective annual long-term follow-up beyond 5 years to collect basic information on the events of interest, while in BCON a one-off retrospective data collection was done to update follow-up data. To overcome this difference, we analysed invasive locoregional control within 5 years of follow-up only. Finally, comparison between trials was not randomised but driven by institutional practice differences, as reflected in the differing proportional split in fractionation regimens between the trials: 177 (39%) of 456 patients in the BC2001 trial received hypofractionated radiotherapy compared with 229 (70%) of 326 in the BCON trial. Although case-mix differences were included in the modelling, there might be unanticipated effects, such as confounding due to differences in clinical decision making. A randomised, controlled trial comparing both schedules would ideally provide the definitive evidence on the question of optimal radiotherapy schedule, but is unlikely to be feasible given the logistics, numbers of patients, and length of follow-up required. In the absence of such evidence, a meta-analysis of individual patient data from the two largest randomised, controlled trials on bladder preservation seems the best approach.

Shorter treatment protocols have numerous socioeconomic advantages in any health-care system. If evidence of superiority of treatment can be provided, with no difference in long-term side-effects or detriment to the patient experience, the protocol should be adopted as standard of care. Therefore, we recommend 55 Gy in 20 fractions as a standard of care for bladder preservation in patients with locally advanced bladder cancer.

## Data sharing

The authors support the wider dissemination of information from the research related to this Article, and increased cooperation between investigators. Trial data was collected and is being managed, stored, shared, and archived according to standard operating procedures of the relevant clinical trial unit (ICR-Clinical Trials and Statistics Unit [ICR-CTSU], Birmingham Cancer Research UK Cancer Trials Unit, and Mount Vernon Cancer Centre) to ensure the enduring quality, integrity, and utility of the data. Formal requests for data sharing will be considered in line with the relevant clinical trial unit procedures with due regard given to funder and sponsor guidelines. Requests should be sent in writing describing the nature of the proposed research and extent of data requirements. Data recipients are required to enter a formal data sharing agreement that describes the conditions for release and requirements for data transfer, storage, archiving, publication, and intellectual property. Requests will be reviewed by the corresponding and senior authors (AC, RH, PH, NDJ) and as appropriate by the BC2001 and BCON trial management groups in terms of scientific merit and ethical considerations including patient consent. Data sharing will be undertaken if proposed projects have a sound scientific or patient benefit rationale as agreed by the corresponding and senior authors and trial management groups, as appropriate. De-identified individual participant data, together with a data dictionary defining each field in the set, will be made available after approval of the request, as well as supporting documentation as required. Restrictions relating to patient confidentiality and consent will be maintained by aggregating and anonymising identifiable patient data. Additionally, all indirect identifiers that might lead to deductive disclosures will be removed in line with Cancer Research UK Data Sharing Guidelines.
