# Supplementary material for: Hypofractionated radiotherapy in locally advanced bladder cancer: an individual patient data meta-analysis of the BC2001 and BCON trials
Source: Lancet Oncol. 2021 Feb;22(2):246–55. doi: 10.1016/S1470-2045(20)30607-0 (PMC7851111; doi:10.1016/S1470-2045(20)30607-0)
Supplement: Supplementary appendix [file mmc1.pdf]

THE LANCET
Oncology

Supplementary appendix

This appendix formed part of the original submission and has been peer reviewed. We post it as supplied by the authors.

Supplement to: Choudhury A, Porta N, Hall E, et al. Hypofractionated radiotherapy in locally advanced bladder cancer: an individual patient data meta-analysis of the BC2001 and BCON trial. *Lancet Oncol* 2021; **22:** 246–55.

Hypofractionated radiotherapy in muscle-invasive bladder cancer: an individual patient data meta-analysis of the BC2001 and BCON trials

Supplementary material

1 Inclusion and exclusion criteria of the BC2001 and BCON trials

BC2001	BCON
**Inclusion criteria:**<br>• Aged 18 or over<br>• Histologically proven invasive bladder carcinoma (adenocarcinoma, transitional or squamous cell carcinoma)<br>• Localised muscle invasive carcinoma either surgically or by imaging (T2-T4a N0 M0)<br>• Patients with multiple tumours at the time of randomisation were not eligible for the radiotherapy volume randomisation but could be randomised to whole bladder radiotherapy with or without synchronous chemotherapy<br>• WHO performance status of grade 0 to 2<br>• Leucocytes > 4.0x10P9P/L, platelets > 100x10P9P/L<br>• GFR > 25ml/min<br>• Serum bilirubin < 1.5 upper limit of reference range (ULRR) ALT or AST < 1.5 x ULRR<br>• Patient available for long term follow up, and in the opinion of investigator, able to receive a radical course of radiotherapy<br>• Patient's written informed consent<br>• For the quality of life (HRQoL) part of the study, able to understand and complete the HRQoL questionnaire	**Inclusion criteria:**<br>• Age over 18 years<br>• Histologically proven transitional cell carcinoma of the bladder<br>• Muscle invasive carcinoma (Stage T2 or T3) of any grade, high grade (G3) superficial bladder carcinoma (T1) or prostatic invasion (T4a)<br>• Ability to give informed consent<br>• Capable of complying with the use of a closed breathing system delivering carbogen through either a mask or a mouthpiece with nasal clip<br>• Any WHO performance status
**Exclusion criteria:**<br>Patients with any of the following were not eligible for the trial:<br>• Uncontrolled systemic disease which would preclude the patient from the study<br>• Pregnancy<br>• Other malignancy within the previous 2 years (other than adequately treated BCC of the skin or adequately treated in situ carcinoma of the cervix uteri)<br>• Previous malignancy that is likely to interfere with protocol treatment<br>• Inflammatory bowel disease<br>• Previous pelvic radiotherapy<br>• Bilateral hip replacements compromising accurate radiotherapy planning	**Exclusion criteria:**<br>• Squamous or adenocarcinoma of the bladder<br>• Locally advanced T4b carcinoma<br>• The presence of distant metastasis or enlarged pelvic lymph nodes on CT staging scan of the pelvis<br>• Co-existing respiratory disease with reduced respiratory drive which would make a delivery of 95% oxygen contra-indicated<br>• Impaired renal or hepatic function resulting in serum creatinine or bilirubin more than twice the normal range<br>• Ischaemic heart disease or peripheral vascular disease requiring treatment with ACE inhibitors

2 Summary of key features (radiotherapy treatment, baseline and follow-up assessments) in the BC2001 and BCON trials

	BC2001<br>Standard RT arm	BC2001<br>Reduced High dose volume arm	BCON
Staging investigations	Cystoscopy, Biopsy+/-TURBT<br>CT/MR abdomen pelvis<br>Chest XR1		Cystoscopy, Biopsy+/-TURBT<br>CT/MR abdomen pelvis<br>Chest XR
Clinical target volume (CTV)	Planned with empty bladder<br>Bladder plus extravesical bladder tumour	Planned with empty bladder<br>CTV1 Bladder plus extravesical bladder tumour<br>CTV2 Gross tumour volume	Planned with empty bladder<br>Bladder plus extravesical bladder tumour
Lymph node radiotherapy	No		No
CTV to planning target volume (PTV)	1.5cm		1.5cm
Radiotherapy technique	Conventional or conformal<br>3 fields	Conformal<br>2 phase or concomitant boost<br>3 fields	3d Conformal<br>3 or 4 fields
Dose	64Gy in 32fractions (f) over 6.5 weeks or<br>55Gy in 20fractions over 4 weeks	64Gy in 32f or 55Gy in 20f to PTV2<br>80% of dose to PTV1 outside PTV2	64Gy in 32fractions over 6.5 weeks or 55Gy in 20fractions over 4 weeks
Health-Related Quality of life (HRQoL)	Yes		No[2]
Follow up cystoscopy	6 and 9 months post randomisation then annually		6 months post radiotherapy treatment then 6 monthly to 5 years
Follow up imaging	Chest X-Ray 6, 9, 12 months post randomisation then annually<br>CT abdomen/pelvis year 1 and 2 and as clinically indicated		As clinically indicated
Endpoints	**Primary:**<br>Locoregional disease‑free survival (defined as the rate of survival free of recurrence in pelvic nodes or bladder, with data censored at the first sign of metastasis (if this occurred ≥30 days before locoregional failure), a second primary tumor, or death.<br>**Secondary:**<br>Disease-free survival, metastasis-free survival, late toxic effects, change in bladder capacity, and quality of life<br>**Tertiary:**<br>Acute toxic effects; cystoscopic local control at 6, 12 & 24 months, rate of salvage cystectomy; and overall survival.<br>**Exploratory:**<br>Time to invasive locoregional recurrence and death from bladder cancer.		**Primary:**<br>Tumor response assessed cystoscopically from 6 months after treatment<br><br>**Secondary:**<br>Overall survival, local relapse-free survival (defined as as time to tumor recurrence in bladder [muscle invasive lesions only], locoregional failure, or death from any cause), early and late rectum and bladder/urethra adverse effects.

TURBT: transurethral resection of bladder tumour; CT: computer tomography; MR: magnetic resonance; XR: X-rays

1 Chest CT also allowed

2 HRQoL planned in BCON, but data return was sparse and analysis not pursued.

3 Statistical Methods: expanded details

Individual patient data (IPD) were obtained from both trials and combined into one dataset. A study identifier unique to each trial was created. Variables available in both datasets were recoded to common names and definitions. Given that the comparison between fractionation schedules was not randomised, and therefore confounding was likely to be present, a one-stage IPD meta-analysis approach was chosen, which was more flexible to adjust for potential confounders.[1-3] In a one-stage approach, analysis was based on the combined dataset, ensuring that clustering within each trial was preserved.[4] There were differences in baseline data collection, which impacted on adjustment of the confounders in the meta-analysis. Forest plots of fractionation effects for each outcome were used to explore the degree of overlap between the 95% confidence intervals (95%CI) of each trial.

The hypothesis of the study was that the hypofractionated RT schedule 55Gy/20f was non-inferior to 64Gy/32f, both in terms of disease control rate and late toxicity. For each endpoint, non-inferiority would be declared if the upper limit of the 95% confidence interval of the estimated fractionation differences was smaller than the non-inferiority margin.

Crude power calculations of non-inferiority based on the number of patients recruited into each trial were performed. Power was computed assuming there were truly no differences between fractionation schedules. As the meta-analysis involved adjusted estimates of fractionation differences, the power was expected to be higher than the below crude estimates. For the primary endpoint ILRC, with a sample size of 791 patients in the combined dataset, we would have 61% power to conclude that 55Gy/20f is non-inferior to 64Gy/32f, assuming an unadjusted log-rank comparison of the fractionation groups, one-sided 0.025 alpha, similar size of fractionation groups (1:1 ratio), and a non-inferiority margin set at hazard ratio of 1.25. If the 2 year-survival in the 64Gy group was 75% (as in BC2001), this margin corresponds to a 2-year rate in the 55Gy being no worse than 69%. For late toxicity, assuming the proportion of GI/GU grade 3 or more LENT/SOMA toxicity overall was 40% in the 64Gy group (from BC2001), this analysis aimed to show that the results in the 55Gy group were no more than 50%, corresponding to a non-inferiority margin of 10% absolute difference. With 791 patients, one-sided alpha 0.025 and 1:1 ratio between fractionation groups, the study would have 83% power to conclude non-inferiority. However, compliance with LENT/SOM questionnaires was low, so 600 patients with data available would give 71% power to exclude such an absolute difference.

All patients in the BCON and BC2001 trials who received at least one fraction of radiotherapy and for whom data on the fractionation schedule was available were included in the meta-analysis. Summaries of baseline characteristics were tabulated by fractionation schedule. Since patients were not randomised to a fractionation schedule, baseline imbalance was expected and investigated using standardised differences. Any variables with a standardised difference greater than 10% were considered potential confounders and investigated further in the meta-analysis.

Median follow-up and number of events for the time-to-event endpoints ILRC and OS were summarised. For each endpoint, a crude analysis to estimate the relative difference (hazard ratio, HR) between fractionation schedules was first performed in the combined dataset by fitting a stratified Cox proportional hazards model with fractionation schedule as the predictor, a frailty term to account for site clustering and stratifying by trial. The latter incorporated the variability between trials as a fixed factor in the model, specifying trial-specific baseline hazard functions, and assuming proportional hazards within each trial. The frailty term for site was added because fractionation schedules were chosen due to local preferences, therefore it was possible that participants treated at the same hospital were more similar in respect to other factors, including unmeasured ones. An adjusted HR for fractionation effect was fitted using a similar model, but incorporating the trial(s) intervention (whether patients received a concurrent radiosensitiser or not), pre-specified prognostic factors and any variable identified as potential confounder with baseline imbalance (leading to >10% variation in the crude fractionation effect when the potential confounder was added to the model) or showing univariable association (at the 5% level) with the time-to-event endpoint. Pre-specified prognostic factors for ILRC were age, sex, tumour stage, use of neoadjuvant chemotherapy and extent of resection; for OS, age and sex were considered.[5] Assumptions of the model were assessed by graphical assessment of residuals. A likelihood ratio test for heterogeneity of fractionation effect across trials was performed by considering an extended model which included the interaction of fractionation schedule and trial. Under the null hypothesis of no heterogeneity between trials, the likelihood ratio statistic followed approximately a chi-square distribution with number of trials-1 degrees of freedom.[6]

The number of patients experiencing grade three or greater GI/GU toxicity within five years was summarised by fractionation schedule overall, and at each time point. Toxicities reported at or after three months prior to first recurrence

or bladder cancer death were treated as missing to avoid interpreting recurrence symptoms as toxicities. The absolute risk difference (RD) between fractionation schedules in having grade three or higher GI/GU toxicity over five years was estimated using a generalized linear binomial model and a random intercept for centre, to account for clustering within sites.[7] A crude model was first fitted with fractionation schedule and including trial as a fixed effect. Parameters of the model were estimated under the generalised estimating equations (GEE) framework: sandwich estimators of the standard errors were produced assuming an exchangeable structure for the working correlation structure, as it assumed equal correlation between any two patients within the same site and that patients from different sites are independent. These estimates were corrected by a sampling correction factor of $J/(J-p-1)$ (J is the number of centres and p is the number of variables in the model) to account for the small number of centres in the data.[8] In the adjusted analysis, we also included the trial(s) intervention, age, sex, and any confounders that were identified as imbalanced at baseline, or associated to the toxicity endpoint in univariate analyses. Heterogeneity between trials was explored considering an interaction effect between fractionation schedule and trial.

Pre-planned subgroup analyses included exploring the fractionation effect within trial and within patients who received radiotherapy alone: a 1% significance level was used in these analyses.

The effect of fractionation schedule on HRQoL was explored in the BC2001 trial only, employing similar methods as used for the trial's HRQoL substudy.9 FACT-BL scores were summarised at baseline, end of trial, 1 and 5 years. Mean difference between fractionation schedules in change from baseline at end of treatment and at one year for the Total (Total), bladder cancer specific (BLCS) and Trial Outcome Index (TOI, sum of BLCS plus physical and functional sub-scales) scores were estimated by analysis of covariance (ANCOVA) regression models, adjusting for trial intervention, baseline score, age, sex, stage and grade. A 1% significance level and corresponding 99% confidence intervals was used to account for multiple time points and subscales.

The risk of bias in the two trials included in the meta-analysis was assessed using a tool developed by the Cochrane collaboration.[10]. Because both trials were unblinded, this was thought to have a potential impact on outcome assessment and reporting. However, the intervention under investigation in this analysis is not the same as for either trial and hence unblinding of randomised treatment is unlikely to bias the effect of fractionation schedule. Therefore, the risk would be judged as low-risk in terms of this analysis. Patients were not randomised to fractionation schedule, so it was expected that fractionation groups would be unbalanced with respect to both subject- and centre-level variables within trial and that confounding may be present. This was accounted for in the analysis by adjusting for the relevant covariates.

References

1. Stewart GB, Altman DG, Askie LM, Duley L, Simmonds MC, Stewart LA. Statistical analysis of individual participant data meta-analyses: a comparison of methods and recommendations for practice. PLoS ONE 2012; **7**(10): e46042.
2. Thomas D, Radji S, Benedetti A. Systematic review of methods for individual patient data meta- analysis with binary outcomes. *BMC Med Res Methodol* 2014; **14**: 79.
3. Riley RD, Lambert PC, Abo-Zaid G. Meta-analysis of individual participant data: rationale, conduct, and reporting. *Bmj* 2010; **340**: c221.
4. Abo-Zaid G, Guo B, Deeks JJ, et al. Individual participant data meta-analyses should not ignore clustering. *J Clin Epidemiol* 2013; **66**(8): 865-73 e4.
5. Huddart R, Porta N, Lewis R, et al. Prognostic factors in muscle invasive bladder cancer patients receiving radiotherapy with or without chemotherapy in the BC2001 trial(CRUK/01/004). *European journal of cancer* 2015; **51**(Suppl 3): S475 #2507.
6. Smith CT, Williamson PR, Marson AG. Investigating heterogeneity in an individual patient data meta-analysis of time to event outcomes. *Stat Med* 2005; **24**(9): 1307-19.
7. Pedroza C, Thanh Truong VT. Performance of models for estimating absolute risk difference in multicenter trials with binary outcome. *BMC Med Res Methodol* 2016; **16**(1): 113.
8. Mancl LA, DeRouen TA. A covariance estimator for GEE with improved small-sample properties. *Biometrics* 2001; **57**(1): 126-34.
9. Huddart RA, Hall E, Lewis R, et al. Patient-reported Quality of Life Outcomes in Patients Treated for Muscle-invasive Bladder Cancer with Radiotherapy +/- Chemotherapy in the BC2001 Phase III Randomised Controlled Trial. European Urology 2020; 77(2): 260-8.
10. Higgins J, Green SE. Cochrane Handbook for Systematic Reviews of Interventions Version 5.1.0 [updated March 2011]. The Cochrane Collaboration. Available from www.handbook.cochrane.org.

4 Analysis of Invasive Loco-regional Control

Table S1. Fractionation effect (55Gy/20f vs 64Gy/32f) in Invasive Loco-Regional Control - crude and adjusted Cox proportional hazards models

	**Crude**				**Adjusted**			
	**64Gy Ev/pts**	**55Gy Ev/pts**	**HR (55Gy to 64Gy ref)**	**95% CI**	**64Gy Ev/pts**	**55Gy Ev/pts**	**HR (55Gy to 64Gy ref)**	**95% CI**
**BC2001**[1]	67/279	34/177	0.82	(0.54, 1.24)	67/275	31/174	0.67	(0.42, 1.06)
**BCON**[2]	39/97	78/229	0.85	(0.58, 1.24)	38/94	73/217	0.77	(0.50, 1.19)
**Combined one-stage IPD meta-analysis**[3]	106/376	112/406	0.83	(0.63, 1.10)	105/369	104/391	0.71	(0.52, 0.96)
Subgroups:								
Received Radiotherapy only[4]	68/216	69/219	0.84	(0.59,1.21)	68/213	64/209	0.72	(0.49,1.05)
Received RT + radiosensitiser[4]	38/160	43/187	0.81	(0.51, 1.27)	37/156	40/182	0.68	(0.42, 1.11)

HR – hazard ratio, CI – confidence interval

[1] *Adjusted for age, sex, randomised treatment, extent of resection, tumour stage, residual mass after resection and neoadjuvant chemotherapy*

[2] *Adjusted for age, sex, randomised treatment, extent of resection, tumour stage and haemoglobin*

[3] *Adjusted for age, sex, , randomised treatment, extent of resection, tumour stage, haemoglobin and neoadjuvant chemotherapy; model stratified by trial and random effect for centre*

[4] *Adjusted for age, sex, extent of resection, tumour stage, haemoglobin and neoadjuvant chemotherapy; model stratified by trial and random effect for centre*

Table S2. Combined one-stage IPD meta-analysis model for Invasive Loco-Regional Control – full adjusted Cox model

**Variable**		**N. events**	**N. patients**	**HR**	**95% CI**
Fractionation	55Gy	104	391	0.71	(0.52, 0.96)
Sex	Female	43	147	0.97	(0.68, 1.37)
Age (years)	Mean (SD)	209	760	1.02	(1.00, 1.04)
Randomised treatment	RT + intervention	77	338	0.65	(0.49, 0.87)
Tumour stage	3	39	125	1.21	(0.84, 1.75)
	4	10	26	1.78	(0.93, 3.42)
Extent of resection	Complete	90	383	0.80	(0.55, 1.18)
	Partial	77	245	1.10	(0.75, 1.61)
Neoadjuvant chemo	Yes	22	132	0.62	(0.37, 1.05)
Haemoglobin (g/dl)	Mean (SD)	209	760	0.86	(0.79, 0.93)

Between-hospital variance= 0.005, Intra-class correlation coefficient=0.03
Likelihood ratio test for heterogeneity in fractionation effect across trials: χ^2=0.066, p=0.80

Table S3. Preliminary analyses of Invasive Loco Regional Control to select variables to adjust for the fractionation effect in full model above

		**Univariable analysis (variable vs outcome)***			**Fractionation effect when adjusted for potential confounders***	
**VARIABLES**	**Categories**	**HR**	**95% CI**	**Wald P-value**	**HR**	**%change**
**Planned total dose schedule**	**55gy 20# vs 64Gy 32#**	**0.83**	**0.630 - 1.103**	**0.2031**	**0.8335**	
Sex	Female vs Male	1.10	0.787 - 1.526	0.5869		
Age at randomisation	year	1.02	1.004 - 1.039	**0.0155**		
Radiosensitiser added to RT?	RT+radiosensitiser vs RT alone	0.67	0.505 - 0.878	**0.0040**		
RT intervention	RHDVRT vs stRT	0.68	0.414 - 1.125	0.1339	0.7972	4.4%
Stage	T3 vs T1/T2	1.18	0.835 - 1.681	0.3424	0.8382	-0.6%
	T4 vs T1/T2	1.94	1.050 - 3.581	**0.0344**		
Grade	3 vs 2	0.78	0.546 - 1.100	0.1543	0.7746	7.1%
Extent of tumour resection	Complete resection vs Biopsy	0.81	0.550 - 1.194	0.2871	0.8506	-2.1%
	Incomplete resection vs Biopsy	1.16	0.790 - 1.716	0.4424		
Haemoglobin	g/dl	0.76	0.575 - 1.009	0.0579		
Neoadjuvant therapy?	Yes vs No	0.68	0.430 - 1.090	0.1106		

* Univariable Cox models stratified by trial. Univariable associations were also explored nonparametrically by plotting Kaplan-Meier estimates and performing log-rank tests.
**To assess confounding, fractionation effect estimate obtained from Cox model including fractionation schedule and each of the potential confounders (>10% imbalance at baseline). The magnitude of confounding was <10% for all variables.

5 Analysis of Overall Survival

Table S4. Fractionation effect (55Gy/20f vs 64Gy/32f) in Overall Survival - crude and adjusted Cox proportional hazards models

	Crude				Adjusted			
	**64Gy Ev/pts**	**55Gy Ev/pts**	**HR (55Gy to 64Gy ref)**	**95% CI**	**64Gy Ev/pts**	**55Gy Ev/pts**	**HR (55Gy to 64Gy ref)**	**95% CI**
**BC2001[1]**	200/279	130/177	1.06	(0.85, 1.33)	196/275	127/174	0.93	(0.73, 1.19)
**BCON[2]**	73/97	168/229	0.87	(0.66, 1.15)	71/94	156/217	0.78	(0.58, 1.05)
**Combined one-stage IPD meta-analysis[3]**	273/376	298/406	0.99	(0.83, 1.18)	267/369	283/391	0.87	(0.72, 1.06)
**Subgroups:**								
Received Radiotherapy only[4]	161/216	170/219	1.06	(0.84,1.33)	158/213	160/209	0.92	(0.72, 1.18)
Received RT + radiosensitiser[4]	112/160	128/187	0.91	(0.70, 1.20)	109/156	123/182	0.83	(0.62, 1.11)

HR – hazard ratio, CI – confidence interval

1 Adjusted for age, sex, WHO, randomised treatment, extent of resection, tumour stage, and haemoglobin

2 Adjusted for age, sex, randomised treatment, extent of resection, tumour stage and haemoglobin

3 Adjusted for age, sex, randomised treatment, extent of resection, tumour stage, haemoglobin; model stratified by trial and random effect for centre

4 Adjusted for age, sex, extent of resection, tumour stage, haemoglobin; model stratified by trial and random effect for centre

Table S5. Combined one-stage IPD meta-analysis model for Overall Survival– full adjusted model

**Variable**		**N. events**	**N. patients**	**HR**	**95% CI**
Fractionation	55gy 20# vs 64Gy 32#	283	391	0.87	0.72, 1.06
Sex	Female vs Male	99	147	0.84	0.67, 1.05
Age (years)	1 year	550	760	1.04	1.03, 1.05
Randomised treatment	RT+radiosensitiser vs RT alone	232	338	0.83	0.70, 0.98
Tumour stage	T3 vs T1/T2	97	125	1.13	0.89, 1.43
	T4 vs T1/T2	21	26	1.49	0.95, 2.34
Extent of resection	Complete resection vs Biopsy	277	383	0.89	0.70, 1.14
	Incomplete resection vs Biopsy	178	245	1.08	0.83, 1.39
Haemoglobin (g/dl)	g/dl	550	760	0.89	0.85, 0.94

Between-hospital variance= 0.007, Intra-class correlation coefficient=0.02
Likelihood ratio test for heterogeneity in fractionation effect across trials: χ^2=5·37, p=0·02

Table S6. Preliminary analyses of Overall Survival to select variables to adjust for the fractionation effect in full model above

		**Univariable analysis (variable vs outcome)***			**Fractionation effect when adjusted for potential confounders***	
**VARIABLES**	**Categories**	**HR**	**95% CI**	**Wald P-value**	**HR**	**%change**
**Planned total dose schedule**	**55gy 20# vs 64Gy 32#**	**0.99**	**0.827 - 1.175**	**0.8705**	**0.9855**	-
Sex	Female vs Male	0.97	0.787 - 1.205	0.8091		
Age at randomisation	year	1.03	1.023 - 1.045	**<0.0001**		
Radiosensitiser added to RT?	RT+radiosensitiser vs RT alone	0.82	0.694 - 0.970	**0.0206**		
RT intervention	RHDVRT vs stRT	0.97	0.752 - 1.239	0.7813	0.9614	2.7%
Stage	T3 vs T1/T2	1.11	0.890 - 1.378	0.3605	0.9773	1.1%
	T4 vs T1/T2	1.51	0.978 - 2.318	0.0633		
Grade	3 vs 2	1.04	0.819 - 1.328	0.7345	0.9421	4.6%
Extent of tumour resection	Complete resection vs Biopsy	0.90	0.700 - 1.156	0.4087	0.9940	-0.6%
	Incomplete resection vs Biopsy	1.08	0.832 - 1.401	0.5651		
Haemoglobin	g/dl	0.78	0.657 - 0.923	**0.0039**		
Neoadjuvant therapy?	Yes vs No	0.88	0.687 - 1.121	0.2941		

* Univariable Cox models stratified by trial. Univariable associations were also explored nonparametrically by plotting Kaplan-Meier estimates and performing log-rank tests.
**To assess confounding, fractionation effect estimate obtained from Cox model including fractionation schedule and each of the potential confounders (>10% imbalance at baseline). The magnitude of confounding was <10% for all variables.

6 Analysis of Bladder Specific Survival

Of the 456 BC2001 patients included in the analysis, 230 (50.4%) died due to bladder cancer (49.8% 64Gy, 51.4% 55Gy), 100 (21.9%) died due to other causes (21.9% 64Gy, 22.0% 55Gy). Median follow-up for bladder cancer deaths was 104 months (IQR 71-121), and median follow-up for deaths due to other causes was 135 (IQR 87-NE).

Of the 326 patients in the BCON trial, 144 (44.2%) died due to bladder cancer (51.6% 64Gy, 41.1% 55Gy), 97 (29.8%) died due to other causes (23.7% 64Gy, 32.3% 55Gy). Median follow-up for bladder cancer deaths was 95 months (IQR 60-142), and median follow-up for deaths due to other causes was 131 (73-NE).

In BCON, cause of death was collected while on active follow-up for the study, but not consistently during retrospective data collection of long-term follow-up. For this reason, we have estimated the fractionation effect for bladder cancer specific survival (BCSS) within 10 years (patients alive by 10 years are censored at t=10). A competing risks analysis was performed to analyse (BCSS).

Figure S1 - Cumulative Incidence of bladder-cancer specific mortality (left) and due to other causes (right) by fractionation schedules in BC2001 and BCON trials

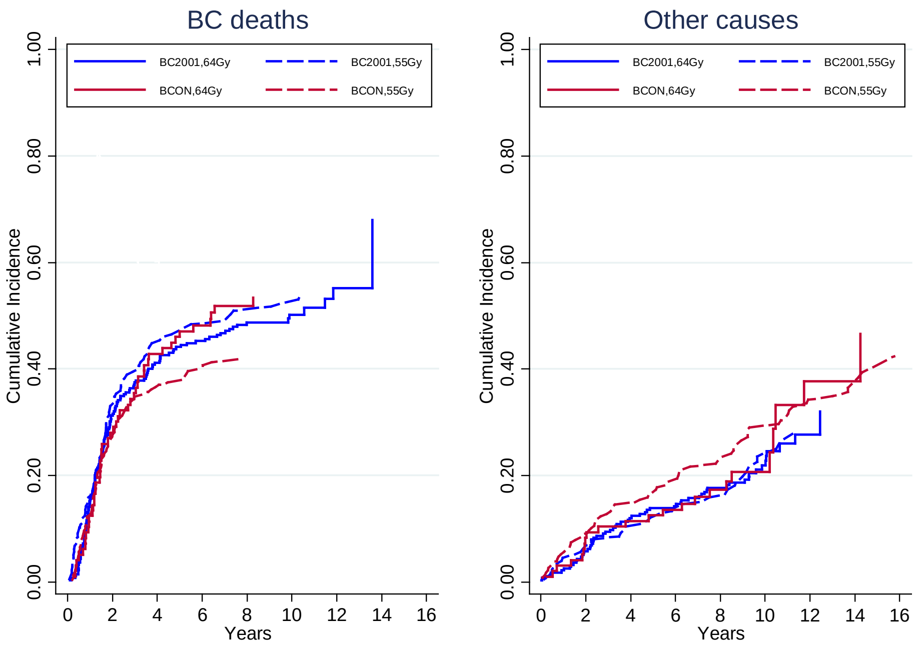

Table S7. Fractionation effect (55Gy/20f vs 64Gy/32f) in Bladder Cancer Specific Survival - crude and adjusted Fine&Gray model

	**Crude**				**Adjusted**			
	**64Gy Ev/pts**	**55Gy Ev/pts**	**HR (55Gy to 64Gy ref)**	**95% CI**	**64Gy Ev/pts**	**55Gy Ev/pts**	**HR (55Gy to 64Gy ref)**	**95% CI**
**BC2001**[1]	139/279	91/177	1.04	(0.76, 1.41)	133/267	82/162	0.93	(0.72, 1.20)
**BCON**[2]	50/97	94/229	0.87	(0.58, 1.30)	50/97	93/228	0.80	(0.52, 1.21)
**Combined one-stage IPD meta-analysis**[3]	189/376	185/406	0.97	(0.76, 1.25)	185/369	174/391	0.83	(0.66, 1.05)

HR – sub-distribution hazard ratio (Fine&Gray model, CI – confidence interval
1 Adjusted for age, sex, WHO, trial intervention, extent of resection, residual mass post-resection, tumour stage, haemoglobin
2 Adjusted for age, sex, trial intervention, extent of resection, tumour stage and haemoglobin
3 Adjusted for trial, age, sex, trial intervention, extent of resection, tumour stage, haemoglobin

7 Analysis of Toxicity

In the BC2001 trial, the proportion of patients with no toxicity data for analysis (either not collected or with any data collected after 3-months prior of a recurrence and thus censored) was greater in the 55Gy group (32.2%) than in the 64Gy group (27.3%). Amongst those with available toxicity data for analysis, 14% recurred (median 60 months), and 65% died (median 79 months); while for patients with no toxicity data available for analysis, 42% recurred (median 5.8 months), and 90% died (median 10.2 months). In the BCON trial, the proportion of patients with all missing or censored toxicity data was similar in the two groups (55Gy 23.6% vs 64Gy 22.7%). Amongst those with available toxicity data for analysis, 27% recurred (median 48 months), and 67% died (median 73 months); while for patients with no available toxicity data for analysis, 64% recurred (median time to recurrence 5.9), and 94% died (median survival time 8.5).

Table S8. Summary of baseline characteristics in BC2001, BCON and the combined dataset in the toxicity analysis population

		**BC2001**				**BCON**				**COMBINED BC2001&BCON**			
**Variable**		**N**	**64Gy (n=203)**	**55Gy (n=120)**	**Std diff (%)**	**N**	**64Gy (n=75)**	**55Gy (n=175)**	**Std diff (%)**	**N**	**64Gy (n=278)**	**55Gy (n=295)**	**Std diff (%)**
Sex	Male	323	171 (84.2)	94 (78.3)	15.2	250	63 (84.0)	141 (80.6)	9	573	234 (84.2)	235 (79.7)	**11.7**
Age (years)	Mean (SD)	323	71.3 (8.7)	71.2 (7.7)	2.3	250	72.1 (8.2)	72.9 (7.9)	9.7	573	71.5 (8.6)	72.2 (7.9)	7.7
Randomised treatment	RT + intervention	323	82 (40.4)	49 (40.8)	1.0	250	42 (56.0)	87 (49.7)	12.6	573	124 (44.6)	136 (46.1)	3.0
Tumour stage	1	323	1 (0.5)	0 (0.0)	47.5	250	11 (14.7)	11 (6.3)	36	573	12 (4.3)	11 (3.7)	**40.2**
	2		184 (90.6)	89 (74.2)			53 (70.7)	120 (68.6)			237 (85.3)	209 (70.9)	
	3		15 (7.4)	28 (23.3)			9 (12.0)	38 (21.7)			24 (8.6)	66 (22.4)	
	4		3 (1.5)	3 (2.5)			2 (2.7)	6 (3.4)			5 (1.8)	9 (3.1)	
Grade	1	322	1 (0.5)	0 (0.0)	10.6	250	0 (0)	0 (0)	6.4	572	1 (0.4)	0 (0.0)	9.2
	2		28 (13.8)	15 (12.6)			12 (16.0)	24 (13.7)			40 (14.4)	39 (13.3)	
	3		174 (85.7)	104 (87.4)			63 (84.0)	151 (86.3)			237 (85.3)	255 (86.7)	
Extent of resection	Biopsy/ Not resected	320	15 (7.5)	14 (11.8)	36.1	243	18 (24.0)	49 (29.2)	15.4	563	33 (12.0)	63 (22.0)	**40.2**
	Complete		138 (68.7)	61 (51.3)			33 (44.0)	62 (36.9)			171 (62.0)	123 (42.8)	
	Partial		48 (23.9)	44 (36.9)			24 (32.0)	57 (33.9)			72 (26.0)	116 (35.2)	
Neoadjuvant chemo	Yes	323	51 (25.1)	43 (35.8)	23.4	250	0 (0.0)	0 (0.0)	0	573	51 (18.4)	43 (14.6)	**10.2**
Haemoglobin (g/dl)	Mean (SD)	323	13.2 (1.8)	12.7 (1.8)	28.5	247	14.0 (1.5)	13.7 (1.5)	17.7	570	13.4 (1.8)	13.3 (1.7)	6.6

Std diff: standardised difference (in %)

Table S9. Fractionation effect (55Gy/20f vs 64Gy/32f) in toxicity - crude and adjusted crude and adjusted binary models estimating the average difference between fractionation groups in absolute risk of experiencing a grade 3/4 bladder or rectum toxicity within 5 years after treatment

	**Crude**				**Adjusted**			
	**64Gy Ev/pts**	**55Gy Ev/pts**	**%Risk difference (55Gy – 64Gy)**	**95% CI**	**64Gy Ev/pts**	**55Gy Ev/pts**	**%Risk difference (55Gy – 64Gy)**	**95% CI**
**BC2001**[1]	62/203	31/120	-4.79	(-15.06, +5.47)	62/203	31/120	-5.24	(-15.78, +5.30)
**BCON**[1]	27/75	66/175	-0.84	(-15.39, +13.71)	27/75	66/175	-0.79	(-17.84, +16.27)
**Combined one-stage IPD meta-analysis**[2]	89/278	97/295	-2.88	(-11.15, +5.39)	89/278	97/295	-3.37	(-11.85, +5.10)
**Subgroups:**								
Received Radiotherapy only[3]	57/154	46/159	-10.81	(-22.16, +0. 55)	57/154	46/159	-12.51	(-23.84, -1.19)
Received RT + radiosensitiser[3]	32/124	51/136	+6.67	(-5.42, +18.76)	32/124	51/136	+7.32	(-5.03, +19.67)

CI – confidence interval; IPD: Individual Patient Data

1 Adjusted for age, sex, and randomised treatment

2 Adjusted for age, sex, randomised treatment and trial; randomised intercept for centre

3 Adjusted for age, sex and trial

Table S10. Combined one-stage IPD meta-analysis model for late toxicity – full adjusted model

**Variable**		**% Risk Difference**	**95% CI**
Fractionation	**55gy 20# vs 64Gy 32#**	-3.37	-11.85, +5.10
Sex	Female vs Male	+13.90	+2.52, +25.27
Age (years)	year	-0.09	-0.59, +0.41
Trial intervention	RT+radiosensitiser vs RT alone	-1.40	-9.43, +6.63,
Trial	BCON vs BC2001	+9.05	+0.30, +17.81

Intraclass correlation coefficient: 0.031
Trial heterogeneity: - test for interaction p=0.54

Table S11. Preliminary analyses of late toxicity to select variables to adjust for the fractionation effect in full model above

		**Univariable analysis (variable vs outcome)***			**Fractionation effect when adjusted for potential confounders***	
**VARIABLES**	**Categories**	**OR**	**95% CI**	**P-value**	**OR**	**%change**
**Planned total dose schedule**	**55gy 20# vs 64Gy 32#**	0.9087	0.626 - 1.319	0.6147	0.9087	0.0%
Sex	Female vs Male	1.8684	1.207 - 2.891	**0.0050**	0.8783	3.3%
Age at randomisation	year	0.9995	0.978 - 1.021	0.9634		
Radiosensitiser added to RT?	RT+radiosensitiser vs RT alone	0.9151	0.641 - 1.305	0.6244		
RT intervention	RHDVRT vs stRT	0.8649	0.489 - 1.529	0.6178		
Stage	T3 vs T1/T2	1.4224	0.891 - 2.271	0.1399	0.9167	-0.9%
	T4 vs T1/T2	0.8351	0.256 - 2.721	0.7649		
Grade	3 vs 2	0.8827	0.536 - 1.454	0.6239		
Extent of tumour resection	Complete resection vs Biopsy	1.4940	0.867 - 2.574	0.1482	0.9163	-0.8%
	Incomplete resection vs Biopsy	1.4373	0.815 - 2.535	0.2102		
Haemoglobin	g/dl	0.7678	0.528-1.117	0.168		
Neoadjuvant therapy?	Yes vs No	0.8578	0.501 - 1.469	0.5766	0.8878	2.3%

* Univariable logistic models for each variable adjusting by trial as a fixed effect
**To assess confounding, fractionation effect estimate obtained from logistic model including fractionation schedule, trial and each of the potential confounders (>10% imbalance at baseline, Table S8). The magnitude of confounding was <10% for all variables.

8 Analysis of Health-Related Quality of life

Table S12. BC2001: General FACT-BL scores per subscale and timepoint, by fractionation schedule

	Baseline			EOT			1 year			5 years		
	N	Median	Q1-Q3	N	Median	Q1-Q3	N	Median	Q1-Q3	N	Median	Q1-Q3
**55Gy/20f**												
BLCS	165	34	29-38	126	26	22-33	87	34	30-38	33	34	30-38
TOTAL	167	123	106-134	126	114	90-125	86	125	111-135	33	129	115-137
TOI	165	79	70-87	124	69	52-79	86	80	70-87	33	83	71-87
EWB	166	20	17-22	127	21	18-24	86	22	19-23	33	23	19-24
FWB	167	21	17-26	125	18	13-23	86	21	17-25	34	24	14-27
SWB	165	25	22-27	125	25	21-28	87	25	22-28	32	24	20-28
PWB	168	25	21-27	127	23	17-26	87	26	22-27	34	26	21-27
**64Gy/32f**												
BLCS	256	35	29-39	223	31	25-35	155	35	31-39	76	35	31-37
TOTAL	254	125	109-133	223	116	99-131	154	127	116-138	74	127	116-135
TOI	253	81	69-88	222	73	60-84	154	83	72-92	74	82	76-89
EWB	254	20	17-22	225	21	19-23	156	21	19-23	75	22	20-24
FWB	254	21	17-25	225	20	14-24	156	23	17-26	75	22	18-26
SWB	250	25	22-28	220	24	22-27	155	25	21-27	73	24	21-27
PWB	255	25	22-27	224	24	20-26	155	26	24-27	74	26	24-28

BLCS= Bladder cancer subscale; EWB=Emotional well-being; FWB= Functional well-being; SWB=Social well-being; PWB= Physical well-being; TOI=Trial Outcome Index (PWB+FWB+BLCS)

Figure S2. Health-Related Quality of Life in BC2001: mean change from baseline (with 99% confidence intervals) in Bladder Cancer Specific Scale (BLCS), Trial Outcome Index (TOI) and TOTAL scores (TOI=BLCS+PWB+FWB)

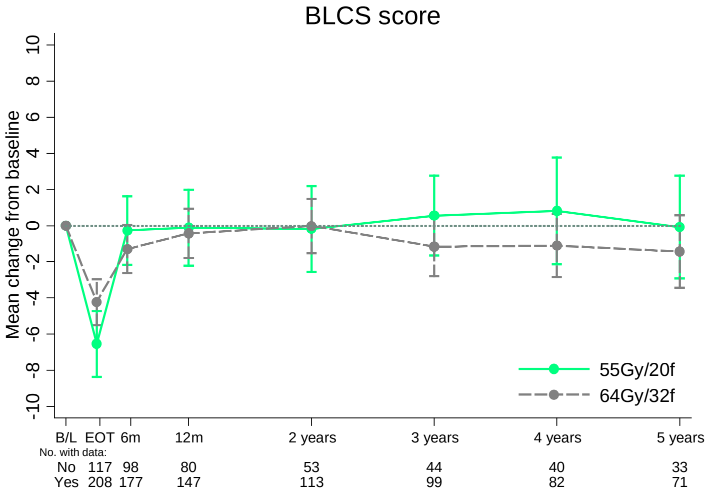

•

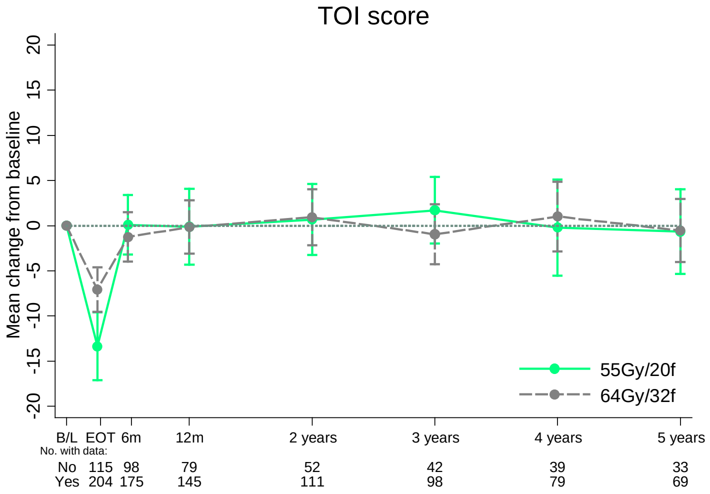

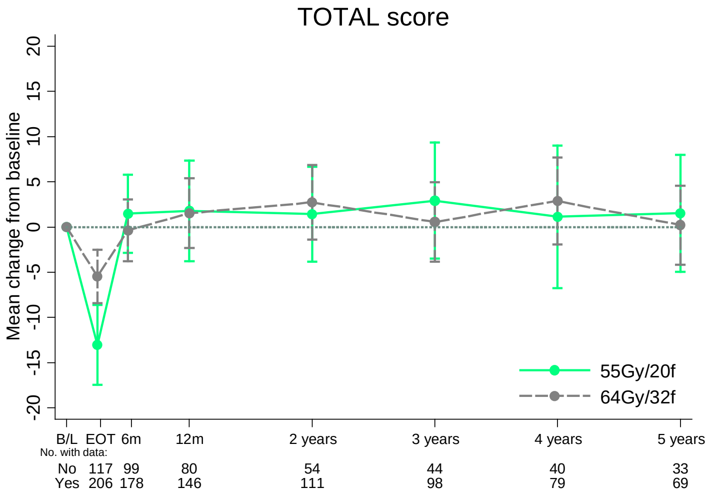

9 Radiobiology of hypofractionation - methods

Conventional and hypo-fractionated treatment regimens can be compared using Biologically Effective Dose (BED) and an equation that includes the effect of treatment time:

$$BED = D * \left[1 + d * {}^{\alpha}/_{\beta}\right] - \gamma(T - Tk)$$

Where D is the total dose, d is the dose per fraction, α/β is a biological parameter that describes the sensitivity to fraction size, γ is a time factor representing the loss of dose per day due to repopulation, T is the overall treatment time and Tk is the kick off time for repopulation.

For normal tissues it is usual to apply the BED formula without the term for repopulation:

$$BED = D * \left[1 + d * {}^{\alpha}/_{\beta}\right]$$

Bladder cancer is considered a rapidly proliferating cancer with an α/β of 10Gy[1] and there is evidence to suggest a loss (γ) of 0.2-0.36 Gy per day after approximately 5 weeks of treatment due to repopulation.[2] Using α/β of 10 Gy without accounting for overall time suggests that 66Gy/32f and 55Gy/20f have Biologically Effective Dose (BED) of 76.8Gy and 70.1Gy respectively. This difference was reduced when a time factor was included, with the maximum reduction for kick-off time (Tk) of 28 days or less. If BED was calculated with γ=0.36 and Tk=28 days, the 64Gy/32f and 55f/20f have BED of 71Gy and 70.1Gy respectively.

Estimates for α/β ratios for late reactions in human bladder range from 3-7.[3] Using a commonly accepted value of 5, the BED for late reactions for 64Gy/32f and 55Gy/20f was 89.6Gy and 85.3Gy respectively, indicating that the longer 2Gy fractionation schedule is marginally 'hotter' than the shorter 20 fraction schedule. It should be noted that using an α/β of 3Gy makes the fractionation schedules equivalent. Also, there was evidence for a time-dependence due to consequential injury from early reactions which reduces the BED for the 64Gy/32f and consequently produces equivalent BED values for late reactions from both fractionation schemes.

1. van Leeuwen CM, Oei AL, Crezee J, et al. The alfa and beta of tumours: a review of parameters of the linear-quadratic model, derived from clinical radiotherapy studies. Radiat Oncol 2018; **13**(1): 96.

2. Maciejewski B, Majewski S. Dose fractionation and tumour repopulation in radiotherapy for bladder cancer. Radiotherapy and Oncology 1991; **21**(3): 163-70.

3. Fowler JF. Sensitivity analysis of parameters in linear-quadratic radiobiologic modeling. Int J Radiat Oncol Biol Phys 2009; **73**(5): 1532-7.
